# Number of Kawasaki Disease Admissions Is Associated with Number of Domestic COVID-19 and Severe Enterovirus Case Numbers in Taiwan

**DOI:** 10.3390/children9020149

**Published:** 2022-01-24

**Authors:** Mindy Ming-Huey Guo, Kuender D. Yang, Shih-Feng Liu, Ho-Chang Kuo

**Affiliations:** 1 Kawasaki Disease Center, Department of Pediatrics, Kaohsiung Chang Gung Memorial Hospital and Chang Gung University College of Medicine, Kaohsiung 83301, Taiwan; mindymhguo@yahoo.com.tw (M.M.-H.G.); liuphysico@yahoo.com.tw (S.-F.L.); 2 Graduate Institute of Clinical Medical Sciences, Chang Gung University College of Medicine, Kaohsiung 83301, Taiwan; 3 Department of Pediatrics, MacKay Memorial Hospital, Taipei 104217, Taiwan; yangkd.yeh@gmail.com; 4 Department of Respiratory Therapy, Kaohsiung Chang Gung Memorial Hospital, Kaohsiung 83301, Taiwan; 5 School of Medicine, Chang Gung University College of Medicine, Taoyuan 33302, Taiwan

**Keywords:** non-pharmaceutical interventions, Kawasaki disease, COVID-19, influenza, enterovirus

## Abstract

**Background:** Non-pharmaceutical interventions (NPIs) introduced in response to the COVID-19 pandemic, including mask-wearing and social distancing, have changed the prevalence of circulating viruses in the community. Since viral infections represent a potential triggering factor for the development of Kawasaki disease (KD), we examined the relationship between KD admission rates and the number of COVID-19, severe influenza, and severe enterovirus infections both before and after the COVID-19 pandemic. **Methods:** We conducted a retrospective study using data obtained from the Chang Gung Research Database (including seven Taiwanese hospitals and more than 10,000 beds) and the Centers for Disease Control in Taiwan from January 2018 to December 2020. We recorded the number of KD admissions, as well as COVID-19, severe influenza, and severe enterovirus infections. **Results:** The numbers of KD admissions, severe enterovirus infections, and severe influenza infections were significantly lower from April to September 2020. The number of KD hospitalizations was positively correlated with the number of domestic COVID-19 cases (*p* = 0.001). A decrease in KD admission numbers was positively correlated with a decrease in severe enterovirus case numbers (*p* = 0.007). **Conclusion:** Our findings provide further evidence that viral infections may be an important trigger factor in the development of KD. Therefore, NPIs may not only prevent transmissible viral infections in children, but also decrease the risk of KD.

## 1. Introduction

Kawasaki disease (KD) is a systemic vasculitis that occurs predominantly in children under the age of five, and is the leading cause of acquired heart disease within this age group. Despite considerable debate regarding the etiology of KD, epidemiological studies indicate that infectious diseases may be a possible trigger for the development of KD. Many studies have documented the seasonal variation in the incidence rate of KD, though the peak months of KD incidence may differ between countries [1]. Possible viral triggers of KD may include human rhinovirus, influenza virus, human bocavirus, enterovirus, coronavirus, and adenovirus [2,3].

In late 2019, a new coronavirus was identified in Wuhan, China, and was subsequently named severe acute respiratory syndrome coronavirus 2 (SARS-CoV-2) which is the virus responsible for COVID-19 (coronavirus disease 2019). In March 2020, the World Health Organization officially declared COVID-19 a global pandemic. Since the beginning of the COVID-19 pandemic, Taiwan has been quite successful at controlling the spread of the disease with a cumulative total of 16,588 confirmed cases of COVID-19 infections and 848 deaths as of November 2021 [4]. Since Taiwan has a current population of approximately 23 million [5], it has one of the lowest per capita infection rates in the world [6]. This achievement is mostly thanks to the rapid response and coordinated effort of Taiwan’s Central Epidemic Command Center [7]. Non-pharmaceutical interventions (NPIs), including mask-wearing mandates in public spaces, social distancing, disinfection of schools and public transportation, and temperature monitoring in schools [7], have not only minimized the total number of pediatric COVID-19 infections in Taiwan, but have also reduced the total number of respiratory infections in children within the same period [8].

Taiwan has one of the highest KD incidence rates in the world, ranking third after Japan and Korea [9]. Such NPIs introduced in response to the COVID-19 pandemic, including mask-wearing mandates and social distancing, may also potentially change the overall prevalence of common circulating viruses in the community. Kang et al. [10] reported in a nationwide study that the KD incidence decreased significantly, by approximately 40%, after the implementation of NPIs in Korea. In Taiwan, our previous report also found a significant decrease of KD incidence, roughly 30%, during the COVID-19 pandemic [11]. Since viral infections are a potential trigger for the development of KD, we wanted to examine the relationship between KD admission rates, number of COVID-19 infections, and traditionally common viral infections (e.g., influenza and enterovirus) both before and after the COVID-19 pandemic.

## 2. Materials and Methods

We conducted a retrospective study using data obtained from the Chang Gung Research Database (CGRD). The CGRD is a de-identified database of medical records collected from Chang Gung Memorial Hospital, which includes seven hospitals from the northeast to the southern part of Taiwan, two of which are tertiary medical centers with a total of more than 10,000 admission beds. Associated with the largest healthcare service provider in Taiwan, the CGRD contains approximately 10% of all inpatient data in Taiwan [12]. We recorded the number of inpatients under the age of 18 who were diagnosed with KD (International Classification of Diseases-10, diagnostic code M30.3) and had received at least one dose of intravenous immunoglobulin (IVIG) during admission from January 2018 to December 2020.

We also reviewed the total number of patients with influenza or enterovirus infections with severe complications, as well as the number of imported and domestic patients diagnosed with COVID-19 from January 2018 to December 2020. This data was obtained from publicly available disease surveillance reports provided by the Centers for Disease Control (CDC) in Taiwan [13]. All confirmed cases of COVID-19 had one or more of the following laboratory criteria: SARS-CoV-2 isolated on viral culture, or positive SARS-CoV-2 RNA detected in a clinical specimen. According to the CDC guidelines in Taiwan, all travelers entering Taiwan from another country must undergo a 14-day period of mandatory quarantine. Imported COVID-19 cases refer to the number of travelers to Taiwan with confirmed COVID-19 diagnosed during or immediately after the quarantine period. Domestic COVID-19 cases refer to all confirmed cases of COVID-19 with no international travel history. Imported COVID-19 case numbers and domestic COVID-19 case numbers are mutually exclusive—the CDC will only identify a COVID-19 patient as being either imported or domestic. As such, imported and domestic COVID-19 numbers are independent of each other and are not confounders.

All confirmed cases of severe influenza were required to have had at least one positive confirmatory test: influenza detected on viral culture, influenza detected via viral PCR, detected influenza antigen, or influenza antibody titers showing a four-fold increase after symptoms of influenza infection appeared. Influenza cases with severe complications were defined as patients with confirmed influenza infections and any of the following complications: myocarditis, pericarditis, invasive bacterial infection, or neurological or pulmonary complications requiring critical care. In addition, all confirmed cases of severe enterovirus were required to have had at least one positive confirmatory test: enterovirus detected on viral culture, enterovirus detected via viral PCR, or positive enterovirus 71 IgM titers. Enterovirus cases with severe complications were defined as patients with confirmed enterovirus infections and any of the following complications: myocarditis, acute myocardial infarction, cardiopulmonary failure, acute hepatitis, or neurological involvement.

To account for seasonality, the data were split into four three-month groupings: January to March, April to June, July to September, and October to December in each year. We then compared the average number of KD admissions, severe influenza, and severe enterovirus infections between the years of 2018–2019 and 2020 using Mann–Whitney U tests. The decrease in the number of admissions for Kawasaki disease, severe influenza, and severe enterovirus cases for each month of 2020 was calculated as follows, using the month of January as an example: (average number of cases per month in January 2018 and January 2019)—(number of cases in January 2020). We also calculated the Spearman correlation coefficient between the number of Kawasaki disease admissions and the number of reported COVID-19 cases, as well as between the number of Kawasaki disease admissions and the number of severe influenza and severe enterovirus cases in the two years prior to the COVID-19 pandemic (2018 and 2019) and after the COVID-19 pandemic (2020). A *p*-value of less than 0.05 was considered statistically significant. This study was approved by the Institutional Review Board of the Chang Gung Medical Foundation (IRB number: 202000966B1 and 20210084B0).

## 3. Results

Average monthly numbers of KD admissions, severe influenza, and severe enterovirus infections were lower from April to September of 2020.

We first examined the number of KD admissions from January 2018 to December 2020 in the CGRD database and found that the annual number of KD admissions from 2018 to 2019 were stable, but decreased in 2020. We also examined CDC monthly reports and recorded the number of reported cases of influenza and enterovirus infection with severe complications in children aged nine years and younger from January 2018 to December 2020 [13]. The annual reported numbers of severe influenza and severe enterovirus infections were similar between the years of 2018 and 2019, but decreased in 2020.

**Figure 1:**
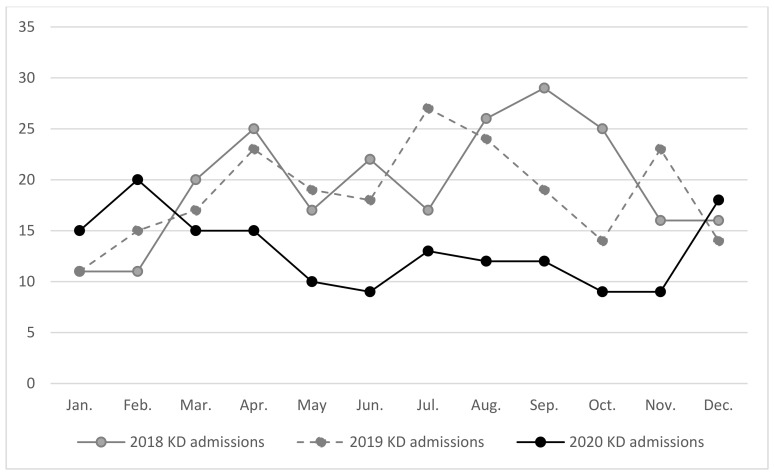
Number of Kawasaki Disease Admissions per Month From 2018 to 2020.

**Figure 2:**
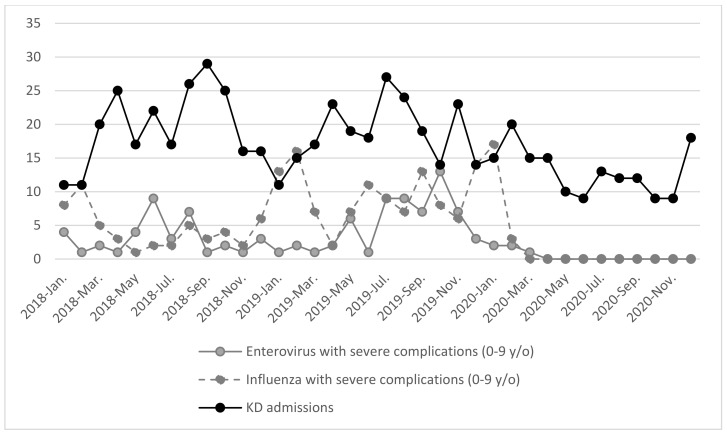
Comparison of KD Admission Numbers, Severe Enterovirus Case Numbers, and Severe Influenza Case Numbers.

As the incidence rates of KD, influenza, and enterovirus infections are seasonal in nature, we separated the data into the following three-month groupings: January to March, April to June, July to September, and October to December of each year. We found that the average monthly number of KD admissions were significantly lower from April to September of 2020 when compared to 2018 and 2019 (Table 1 and Figure 1). Similarly, the average monthly numbers of severe enterovirus infections were lower from April to December of 2020 when compared to the same period from 2018 to 2019. The average monthly numbers of severe influenza cases were lower from April to December of 2020 when compared to 2018 to 2019 (Table 1 and Figure 2).

### 3.1. Number of KD Admissions Was Positively Correlated with Number of Domestic COVID-19 Cases and Reported Number of Severe Enterovirus Infections with Severe Complications

**Figure 3:**
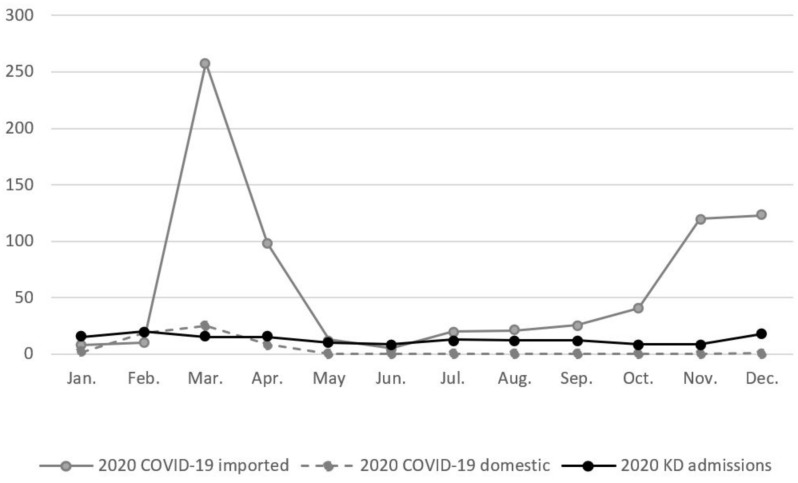
Comparison of Monthly KD and COVID-19 Case Numbers in 2020.

Taiwan had a total of 799 confirmed cases of COVID-19 from January 2020 to December 2020, 743 of which were imported; the remaining 56 cases were the result of domestic transmission. Only five children aged nine years or younger with COVID-19 were reported within this period, although data from the CDC did not specify whether they were imported or domestic [13]. We then compared the number of KD admissions and the number of imported and domestic COVID-19 cases within this period, and found that the number of KD admissions was positively correlated with the number of domestic COVID-19 cases (Spearman correlation coefficient = 0.824, *p*-value = 0.001, Figure 3 and Supplementary Figure S1), but was not associated with the number of imported cases. This means that during 2020, KD admission numbers moved in the same direction as domestic COVID-19 numbers. In other words, we found that in 2020 lower KD admission numbers were correlated with lower domestic COVID-19 numbers, possibly reflecting decreased infectious disease transmission during this period due to the adoption of NPIs.

### 3.2. Number of Kawasaki Disease Admissions Were Associated with Number of Severe Enterovirus Cases in 2020

To determine whether there was an association between the number of KD admissions and the number of severe influenza and enterovirus infections in the periods before and after the COVID-19 pandemic, we split our data into two time periods: the pre-COVID-19 period (i.e., 2018 and 2019) and the post-COVID-19 period (i.e., 2020). In the “pre-COVID-19” period from 2018–2019, we found that KD admission numbers were not correlated with severe enterovirus infection numbers (Spearman correlation coefficient = 0.191, *p* = 0.372), but were negatively correlated with severe influenza numbers (Spearman correlation coefficient = −0.437, *p* = 0.033). The plots in Figure 2 show the corresponding trends between cases of enterovirus, influenza, and KD until November 2019. In the “post-COVID-19” period (i.e., 2020), we found that KD admission numbers were correlated with severe enterovirus infection numbers (Spearman correlation coefficient = 0.603, *p* = 0.038) but were not associated with the number of severe influenza infections (Spearman correlation coefficient = 0.508, *p* = 0.092). The plots in Figure 2 show the corresponding trends between cases of enterovirus, influenza, and KD after January 2020.

**Figure 4:**
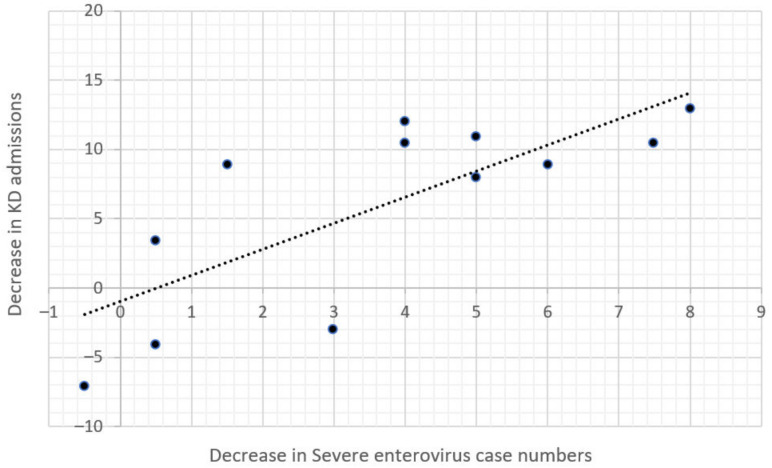
Correlation Between the Decrease in KD Admissions and Severe Enterovirus Numbers in 2020.

**Figure 5:**
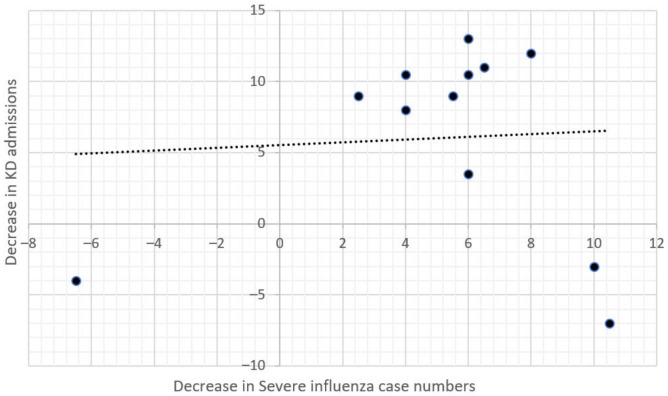
Correlation Between the Decrease in KD Admissions and Severe Influenza Numbers in 2020.

We also found that the decrease in KD admission numbers in 2020 was significantly associated with the decrease in severe enterovirus case numbers (Spearman correlation = 0.732, *p* = 0.007, Figure 4) but was not associated with the decrease in severe influenza case numbers (Spearman correlation = −0.130, *p* = 0.969, Figure 5). This may partly be explained by seasonality, as both KD and enterovirus peak in the summer months, while influenza peak in the winter. Additionally, KD admission numbers were not correlated with severe enterovirus case numbers prior to January 2020, but were correlated with enterovirus case numbers after January 2020, which is probably due to a decrease in overall infection rates after the adoption of NPIs.

## 4. Discussion

Taiwan reported its first case of COVID-19 on 21 January 2020. From January to April of 2020, Taiwan experienced its first wave of COVID-19 infections, peaking in the months of March and April. Infections then began to drastically decline in May of 2020, following a series of policies that included mask rationing and public mask mandates beginning on 6 February 2020, a delayed return to all schools of elementary level and above for two weeks (extending the winter vacation from 21 January to 25 February 2020, although daycare centers remained open during this period), and barring the entry of foreign nationals on 19 March 2020. In our study, we found that the numbers of KD admissions, as well as severe influenza and severe enterovirus infections all declined in 2020 when compared to previous years. The number of KD admissions was significantly lower from the months of April to September of 2020. Likewise, severe enterovirus and influenza infection numbers decreased within the same period. We also observed that the number of KD hospitalizations was positively correlated with the number of domestic COVID-19 cases, but not associated with the number of imported cases. This finding suggests that the reduction in KD hospitalizations may be due to stringent public health policies and other NPIs and the subsequent reduction in transmission of infectious diseases. In our dataset, we found that both severe influenza and severe enterovirus infections were statistically lower from April to September 2020. Moreover, we found that the decline in KD admission numbers was positively correlated with the decline in severe enterovirus infections, but not with the decline in severe influenza infections.

Our results are similar to the findings found in countries where COVID-19 infection rates have been relatively low. Since the pandemic began, the cumulative rate of COVID-19 infections in Taiwan has been very low (70.031 per 100,000 people) [4,5]. In other countries where COVID-19 infection rates are similarly low, such as Japan, South Korea, and Finland (cumulative COVID-19 incidence rates of 1363.841, 762.093, and 3231.09 per 100,000 people, respectively) [6], KD incidence rates declined following the implementation of public health measures in 2020. In Japan, where a state of emergency was declared from 7 April–25 May 2020, and school closures were enacted between 2 March–31 May 2020, incidence rates of KD began to decline beginning in March in 11 hospitals in the Yamanashi prefecture [14], in April in 18 hospitals in the Kobe area [15], and in June in six hospitals in Fukuoka [16]. In both Finland and South Korea, KD incidence rates declined after the implementation of school closures. Datasets from the studies in South Korea and Finland were both obtained from nationwide health registries [10,17].

In countries where the rate of COVID-19 infections has been higher, such as the United States, France, and Italy (cumulative COVID-19 incidence rates of 14,048.689, 10,809.841 and 8107.502 per 100,000 people, respectively) [6], reports regarding KD incidence rates during the COVID-19 period have been more mixed. In a study of 51 pediatric hospitals in the United States, the overall annual number of KD admissions was lower in 2020 when compared to the previous four years. However, a peak in KD admissions occurred in May of 2020 immediately after the initial wave of COVID-19 infections in the United States. Within this study, of the 1383 patients diagnosed with KD in 2020, 108 (8%) were also positive for COVID-19 infection [18]. In Paris, France, a single-center retrospective study recorded a marked increase of KD cases beginning two weeks after the first wave of COVID-19 infections in the country; eight out of the ten cases of KD diagnosed from April 15 to 20 May 2020 also tested positive for SARS-CoV-2 infection [19]. A spike of ten Kawasaki-disease-like cases was also observed following the initial wave of COVID-19 infections in a single-center report from Italy, with two of the patients testing positive for SARS-CoV-2 infection [20]. In all three of these studies, patients diagnosed with KD were on average older, more likely to have cardiac abnormalities such as myocarditis and coronary artery aneurysms, and were also more likely to require intensive care and adjunctive therapy in addition to IVIG treatment [18,19,20]. Because these spikes in KD incidence rates appeared to immediately follow a surge in local COVID-19 infection numbers, it seems reasonable to postulate that SARS-CoV-2 may be an infectious trigger leading to the development of KD. Another more likely explanation may be that these spike in cases reflect a clinical overlap between KD and multisystem inflammatory syndrome in children (MIS-C), an emerging disease entity occurring in children with COVID-19 infections. According to the Centers for Disease Control and Prevention health advisory, the case definition of MIS-C includes the following: fever, laboratory evidence of inflammation, severe illness requiring hospitalization, and multisystem (>2) organ involvement in a patient with confirmed SARS-CoV-2 infection under the age of 21 years [21]. Patients with MIS-C often present with KD-like symptoms. In a systemic review of 655 patients with MIS-C, rash and conjunctivitis were the most common KD-like symptoms (58% and 40%, respectively), followed by fissured lips (23%), swelling of the hands and feet (13%) and cervical lymphadenitis (4%) [22]. Of note, no cases of MIS-C had been reported in Taiwan as of November 2021.

In this study, we also found that the decline in KD admission numbers was positively correlated with the decline in severe enterovirus infections but not with severe influenza infections. This finding can be partially explained by the seasonality and peak incidence months of these three diseases. In Taiwan, KD incidence rates peak from April to June of each year and then decline during the winter months [23]. This phenomenon coincides with the timing of enterovirus infections, which have higher infection rates in the warmer months, then decreasing in the winter months [24]. In addition, severe enterovirus infections are more likely to occur in children younger than six years old [24], which is also the peak age range for KD. In contrast, influenza infection rates usually peak during the winter months and decline in the summer. Our findings are similar to a Korean study that found a significant correlation between the monthly incidence of KD and enterovirus infections [2]. A population-based study utilizing data from Taiwan National Health Insurance found that children aged three to five years who were infected with enterovirus were 56% more likely to develop KD when compared with those who were not infected [25]. Furthermore, in a study of 226 patients with KD, 16.8% were positive for enterovirus, compared to only 4.4% of healthy controls [3].

One limitation of our study is that we only included severe influenza and severe enterovirus case numbers in our analysis. According to infectious disease policy in Taiwan, all medical institutions are required by law to report the number of severe influenza and severe enterovirus cases to the CDC, and to provide specimens from all cases to the CDC for confirmatory testing. Therefore, we believe that severe influenza and severe enterovirus infection numbers are a more accurate reflection of overall disease burden when compared to admission numbers or out-patient clinic numbers, which refer to number of times the disease billing codes for influenza or enterovirus are reported to the National Health Insurance, and are not further confirmed by laboratory testing.

In conclusion, we found that KD admission rates declined during the COVID-19 pandemic period in Taiwan. Additionally, KD admission rates were positively correlated to domestic, but not imported, COVID-19 cases, suggesting that the decline in KD admissions may be attributed to increased public health measures and other NPIs implemented to prevent transmissible infectious disease during this period. Indeed, both severe influenza and severe enterovirus cases in children were lower after April 2020, compared to the same period in previous years. Of note, the decline in KD admission numbers was positively correlated with the decline in severe enterovirus infections, but was not associated with the decline in severe influenza infection. This observation may reflect an overlap in seasonality and the peak age of susceptibility to KD and enterovirus infections. Our findings provide further evidence that viral infections may be an important inciting factor in the development of KD in children. Public health safety measures and NPIs including mask wearing, hand washing, and appropriate social distancing may not only prevent transmissible viral infections in children, but also decrease the risk of KD. However, it is important to note that correlation does not imply causation, and further mechanistic studies are still needed to examine the link between viral infections and the development of KD.

## Figures and Tables

**Table 1:** Comparison of the number of Kawasaki disease admissions, severe influenza infections, and enterovirus infections in the pre-COVID-19 and post-COVID-19 periods.

	Pre-COVID-19 Period (2018–2019)	Post-COVID-19 Period (2020)	*p*-Value
Annual number of KD admissions	2018: 235 2019: 224	2020: 157	
Average KD admissions (per month) ^#^			
Jan.–Mar.	14.17 ± 1.56	16.67 ± 1.67	0.347
Apr.–Jun.	20.67 ± 1.28	11.33 ± 1.86	0.020 *
Jul.–Sep.	23.67 ± 1.93	12.33 ± 0.33	0.020 *
Oct.–Dec.	18.00 ± 1.95	12.00 ± 3.00	0.019 *
Annual number of severe influenza cases	2018: 52 2019: 113	2020: 20	
Average number of severe influenza cases (per month) ^#^			
Jan.–Mar.	10.00 ± 1.67	6.67 ± 5.24	0.439
Apr.–Jun.	4.33 ± 1.59	0.00 ± 0.00	0.018 *
Jul.–Sep.	6.50 ± 1.67	0.00 ± 0.00	0.018 *
Oct.–Dec.	6.67 ± 1.69	0.00 ± 0.00	
Annual number of severe enterovirus cases	2018: 38 2019: 61	2020: 5	
Average number of severe enterovirus cases (per month) ^#^			
Jan.–Mar.	1.83 ± 0.48	1.67 ± 0.33	0.888
Apr.–Jun.	3.83 ± 1.30	0.00 ± 0.00	0.018 *
Jul.–Sep.	6.00 ± 1.34	0.00 ± 0.00	0.017 *
Oct.–Dec.	4.83 ± 1.83	0.00 ± 0.00	0.018 *

Average cases per month are expressed as mean ± standard error (^#^). A *p*-value of less than 0.05 is considered significant (*).

## Data Availability

The data presented in this study are openly available from References [4,13].

## Supplementary Materials

The following supporting information can be downloaded at: https://www.mdpi.com/article/10.3390/children9020149/s1, Figure S1: Correlation Between Kawasaki Disease Admission Numbers and Domestic COVID-19 case numbers in 2020.
