# Supplementary figures and images for: Number of Kawasaki Disease Admissions Is Associated with Number of Domestic COVID-19 and Severe Enterovirus Case Numbers in Taiwan
Source: Children (Basel). 2022 Jan 24;9(2):149. doi: 10.3390/children9020149 (PMC8870605; doi:10.3390/children9020149)

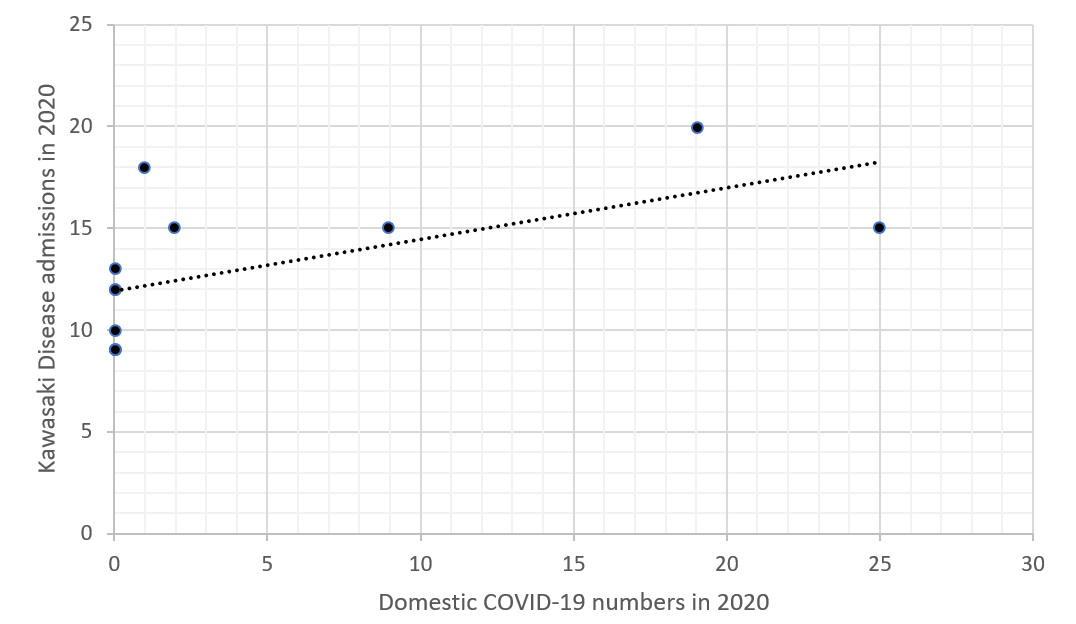

Supplement: Supplementary file 1 [file children-09-00149-s001.zip › children-1510046-supplementary.tif]
